# Development of thermo-responsive polycaprolactone macrocarriers conjugated with Poly(N-isopropyl acrylamide) for cell culture

**DOI:** 10.1038/s41598-019-40242-0

**Published:** 2019-03-05

**Authors:** Linh T. B. Nguyen, Akinlolu O. O. Odeleye, Chih–Yao Chui, Timothée Baudequin, Zhanfeng Cui, Hua Ye

**Affiliations:** 1 Institute of Biomedical Engineering, Department of Engineering Science, University of Oxford, Oxford, OX3 7DQ UK; 2 Present Address: Adaptimmune Limited, 60 Jubilee Avenue, Milton Park, Abingdon, OX14 4RX UK

## Abstract

Poly(N-isopropyl acrylamide) (PNIPAAm) is a well-known ‘smart’ material responding to external stimuli such as temperature. PNIPAAm was successfully conjugated to polycaprolactone (PCL) bead surfaces through amidation reaction. Functionalization steps were characterized and confirmed by Fourier transform infrared spectroscopy, X-ray photoelectron spectroscopy, scanning electron microscopy and Energy Dispersion Spectroscopy. PNIPAAm-conjugated PCL allowed human dermal fibroblast cells (HDF) and mesenchymal stem cells (MSC) to adhere, spread, and grow successfully. By reducing the temperature to 30 °C, more than 70% of HDF were detached from PNIPAAm-conjugated PCL macrocarriers with 85% viability. The cell detachment ratio by trypsin treatment was slightly higher than that induced by reduced temperature, however, cell detachment from PNIPAAm-conjugated macrocarriers by lowering the temperature significantly reduced cell death and increased both cell viability and the recovery potential of the detached cells. HDF attachment and detachment were also observed by Live-Dead staining and phase contrast imaging. The expression of extracellular matrix proteins such as Laminin and Fibronectin was also affected by the trypsinization process but not by the reduced temperature process. Taken together, our results showed that thermo-responsive macrocarriers could be a promising alternative method for the non-invasive detachment of cells, in particular for tissue engineering, clinical applications and the use of bioreactors.

## Introduction

Bio-industries producing vaccines, enzymes, hormones and cytokines require a large number of cells, which are structurally as well as functionally healthy for the intended purposes^1–3^. Apart from the industrial requirements of scaled up cells, clinical application of cell based therapeutic treatments often requires a large number of cells^4,5^. Furthermore, recent advances in stem cell biology and its therapeutic applications have doubled the necessity for scaling up cells. One easy way to expand cells is to use cell carriers in bioreactors where the cells are grown on suspended beads or particles in culture media and benefit from a controlled microenvironment with chemical and mechanical cues^6^. Since the late 70 s, the use of macro- and microcarriers has been popular due to their high surface-area-to-volume ratio that offers large available culture surface for cell expansion while requiring smaller vessels and consumable volumes than flat substrates^7^. However, for therapeutic and clinical applications the harvested and recovered cells should maintain both quantity and quality following hassle-free collection without hindrance^5,8,9^. Thus successful recovery is not only determined by the total amount of cells collected but also by their intact biological properties and recovery potential, which also increases effectiveness in application. Typical enzymatic digestion for recovering cells from macrocarriers is carried out by trypsin, accutase or collagenase either in bench-top lab-scale studies or in bioreactors^10,11^. Although an effective number of cells are collectable by these enzymatic treatments, a major disadvantage is the chance of adversely affecting cellular physiology, altering cellular phenotypic characteristics during passage to passage culturing, and/or affecting the expression of ECM proteins^12–15^.

Recently thermo-responsive microcarriers containing poly (N-isopropyl acrylamide) (PNIPAAm) have gained particular attention because of their ability to propagate and recover cells without physical damage^12^. Thermo-responsive PNIPAAm has the unique feature of being able to change from a ‘random coil’ conformation to a ‘collapsed globular’ conformation, thus creating alteration of the substrate surface from a hydrophilic to a hydrophobic environment depending on the temperature^16^. Simply by changing the temperature whilst harvesting enables quick cell detachment. Instead of conventional harvest protocols which deploy enzymatic treatments, this newly developed technique has been shown not to alter cell physiology, morphology, immunophenotype or osteogenesis of rat bone marrow and human adipose tissue (BM-MSCs and AT-MSCs)^17^. In a previous study from our lab, PNIPAAm-coated thermo-responsive dishes were used to collect cells to fabricate 3D cell sheets of hMSCs in conjugation with PLGA-based electrospun layers by lowering the temperature to 20 °C for 20 minutes^18^. Similarly, Hee Seok Yang *et al*. used PNIPAAm to graft commercially available microcarriers Cytodex-3® which allowed them to collect more hBMMSCs by simply reducing the temperature from 37° to 32 °C^19^. It was also used to entrap and deliver drugs and cells on-demand to other external stimuli such as light signals^20^. It was made possible in particular thanks to the reversibility of the changes in conformation^21^. Thus, its ability to respond to an external stimuli such as temperature has led PNIPAAm to be considered as an ‘intelligent’ or ‘smart’ material.

In the current study, instead of micro-carriers, we have used polycaprolactone (PCL) beads as a macrocarrier substrate and coated the surface with PNIPAAm. Macro-size carriers were chosen for several reasons. Like microcarriers, macrocarriers have high cell density per unit volume with a potential for obtaining highly concentrated cell products compared to flat culture surfaces and bulk scaffolds. Moreover, the ease of production, handling, and maintenance as well as cost effectiveness are main factors for industrial application. In the perspective of their application in complex systems such as bioreactors, macrocarriers are more easily handled and monitored compared to microcarriers that can adhere to walls, are harder to confine and require multiple steps to be isolated and concentrated. Furthermore, macrocarriers made of bulk polymer such as the PCL beads presented here can be used directly, in contrast to microcarriers such as Cytodex© beads which require swelling before cell seeding^22^ which extends the total duration of the process. PCL is a biodegradable and biocompatible polymer that has been approved by the Food and Drug Administration (FDA) for specific applications used in the human body and for cell therapies^23^. Due to the excellent long-term biocompatibility and minimal inflammatory responses in animal models, PCL is a suitable substrate material for cell-supporting macrocarriers^24^. The purpose of this study was to produce thermo-responsive macro-beads suitable for cell harvesting without the use of generally employed proteolytic enzymes in bioreactors and to assess the recovery potential of harvested cells. We constructed thermo-sensitive PCL-PNIPAAm macrocarriers from which Human Dermal Fibroblast cells (HDF) and MSCs could be detached without proteolytic enzyme treatment. We also investigated whether PCL-PNIPAAm altered cell adhesion and growth and whether cell detachment by temperature reduction caused less damage to the cells than trypsin treatment.

## Results

### Characterization of PNIPAAm immobilized on PCL surface

**Figure 1:**
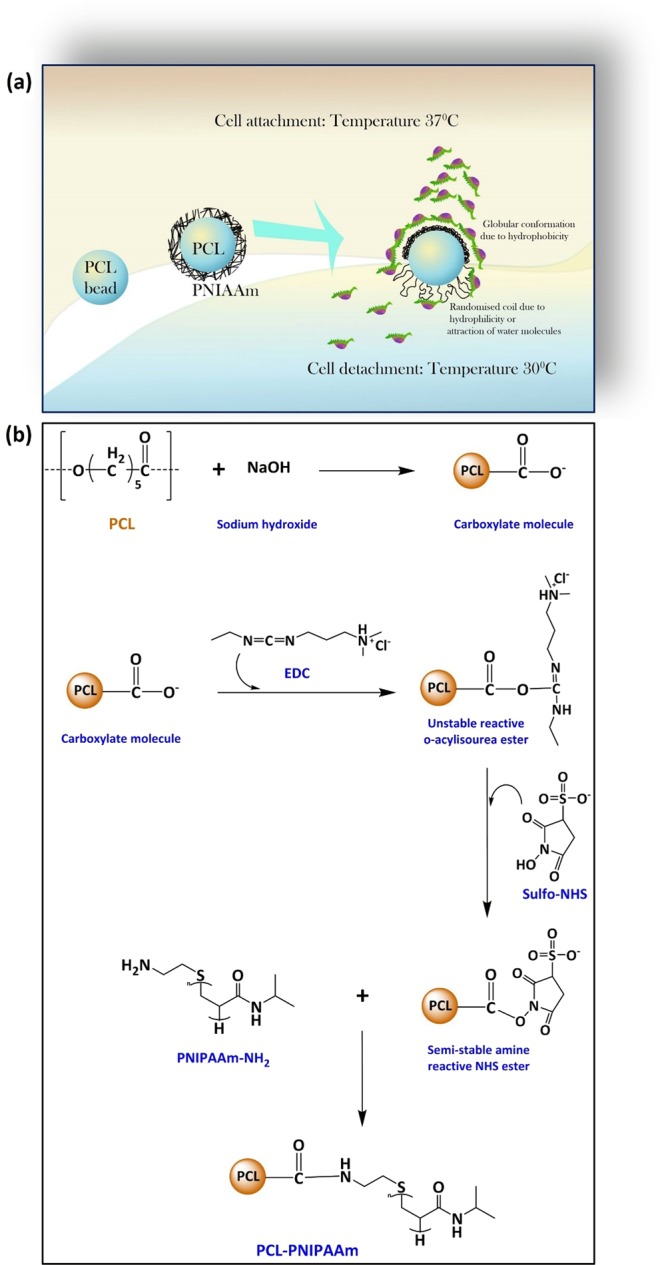
Schematic diagram. Schematic diagram of the thermal response of cells on PNIPAAm immobilized on PCL surfaces (**a**) as well as the reactions to immobilize PNIPAAm on PCL (**b**). At 37 °C the conformation of PNIPAAAm is like a random coil which provides a hydrophobic surface on PCL macrocarriers. When the temperature is dropped down to 30 °C, the conformation changes to globular causing the surface to be hydrophilic in nature. A hydrophobic surface attracts cells whereas a hydrophilic surface repels the cells as shown in figure (**a**). In order to immobilize PNIPAAm on PCL surfaces a simple amidation reaction was carried out following the introduction of both carboxylic and amine groups on PCL surfaces and in PNIPAAm respectively (**b**). The amine group (NH_2_) was introduced to PCL-COO- whereas carboxylic groups on PCL were activated by using EDC/NHS.

Figure 1a shows a schematic diagram of thermo-responsive polymer immobilized onto the surfaces of PCL and the temperature-dependent effect of cell attachment to and detachment from the PCL-PNIPAAm surface. Lowering the temperature to as low as 30 °C caused cellular detachment due to the hydrophobic conformation of PNIPAAm. The scheme of conjugation of PNIPAAm-NH_2_ to PCL is shown in Fig. 1b. Sodium hydroxide (NaOH) treatment formed carboxyl groups, increased hydrophilicity of the PCL beads and also created nanoroughness, which resulted in improved cell-material interactions and increased surface area, which enhanced cell adhesion.

**Figure 2:**
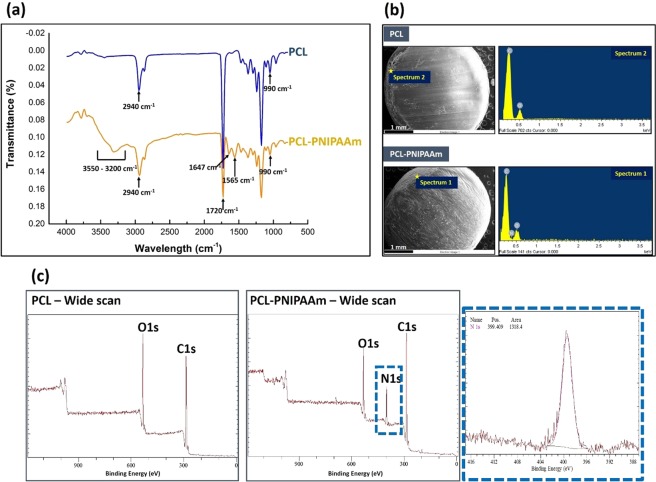
Surface modification and characterization of PNIPAAm conjugated PCL. ATR-FTIR analysis of PCL and PCL-PNIPAAm confirmed the successful conjugation of PNIPAAm to PCL. Several new peaks appeared due to PNIPAAm-NH_2_ introduction onto PCL-COOH (**a**). Surface morphology was observed by scanning electron microscopic images and the EDS profile showed the appearance of a nitrogen peak as the PNIPAAm (**b**). This was further confirmed by XPS spectra scan by the appearance of a N1s peak in PCL-PNIPAAm surfaces (**c**).

Fourier transform infrared (FTIR) spectroscopy, scanning electron microscopy with Energy Dispersion Spectroscopy (SEM-EDS) and X-ray photoelectron spectroscopy (XPS) analyses were used to confirm the conjugation of PNIPAAm-NH_2_ to the surface of PCL beads. The FTIR spectrum in Fig. 2a shows that the wide peak between 3550 and 3200 cm^−1^ belonged to the N-H stretching of the modified PCL beads. The increased peak at 2940 cm^−1^ is attributed to the vibration of aliphatic groups (-CH_2_−)_n_ of the copolymer. The increased intensity of the peak at 1647 cm^−1^ could indicate an amide I bond, arising from C=O stretching and little C-N stretching of PNIPAAm. The peaks at 1565 cm^−1^ corresponded to the amide II bond, arising from N-H bending and C-N stretching of PNIPAAm. This suggested that the conjugation of the PNIPAAm on PCL beads was successful. The surface morphology of unmodified and modified macrocarriers was characterized by SEM and is shown in Fig. 2b. Conjugation of PNIPAAm onto the PCL did not affect the surface morphology of the PCL. The appearance of a nitrogen peak (EDS images) in the PCL-PNIPAAm confirmed that the polymerization was carried out properly.

XPS can determine the chemical composition of a surface’s top to within several nanometers. The appearance of a new N1s signal with binding energy at 400 eV after the immobilization of PNIPAAm in the wide scan XPS spectra, as shown in Fig. 2c, was indicative of successful conjugation of PNIPAAm on the PCL macrocarriers surface. The N1s core-level spectra from PCL-PNIPAAm was curve-fitted with a peak at 399.4 eV attributable to the amino group (-NH_2_). Detection of N and C from C=O bonds meant that PNIPAAm-NH_2_ was present on the surface of PCL beads. The C:O:N ratio results in Table 1 obtained by XPS analysis show that the immobilization amount of PNIPAAm was regulated by changing the feed concentration of the PNIPAAm-NH_2_. The nitrogen content, which was increased from 0.2 g of PNIPAAm-NH_2_ to 2.0 g of PNIPAAm-NH_2_ and then kept unchanged, indicated that the immobilized density was limited, reaching a plateau at 2.0 g of PNIPAAm-NH_2_. This limited density happened due to a strong kinetic hindrance caused by the already deposited polymer chains^25^. Once the surface becomes significantly covered, additional polymer chains have to diffuse through the existing polymer layer to reach the surface before they can be immobilised^26^.

**Table 1:** Characterization of C:O:N ratio on PCL-PNIPAAm by XPS with various amounts of PNIPAAm-NH_2_.

PCL-COO^−^ (g/20 ml of MES)	PNIPAAm-NH_2_ (g/20 ml of MES)	EDC (g/20 ml of MES)	NHS (g/20 ml of MES)	C:O:N ratio
3.0	0	0.46	0.14	6.0:2.0:0
	0.2			6.7:2.0:0
	0.5			6.4:2.4:0
	1.0			6.8:2.7:0.7
	2.0			7.0:2.0:1.0
	3.0			7.3:2.2:1.0
	4.0			7.2:2.5:1.0

### Cell viability, cytotoxicity and proliferation assessment

**Figure 3:**
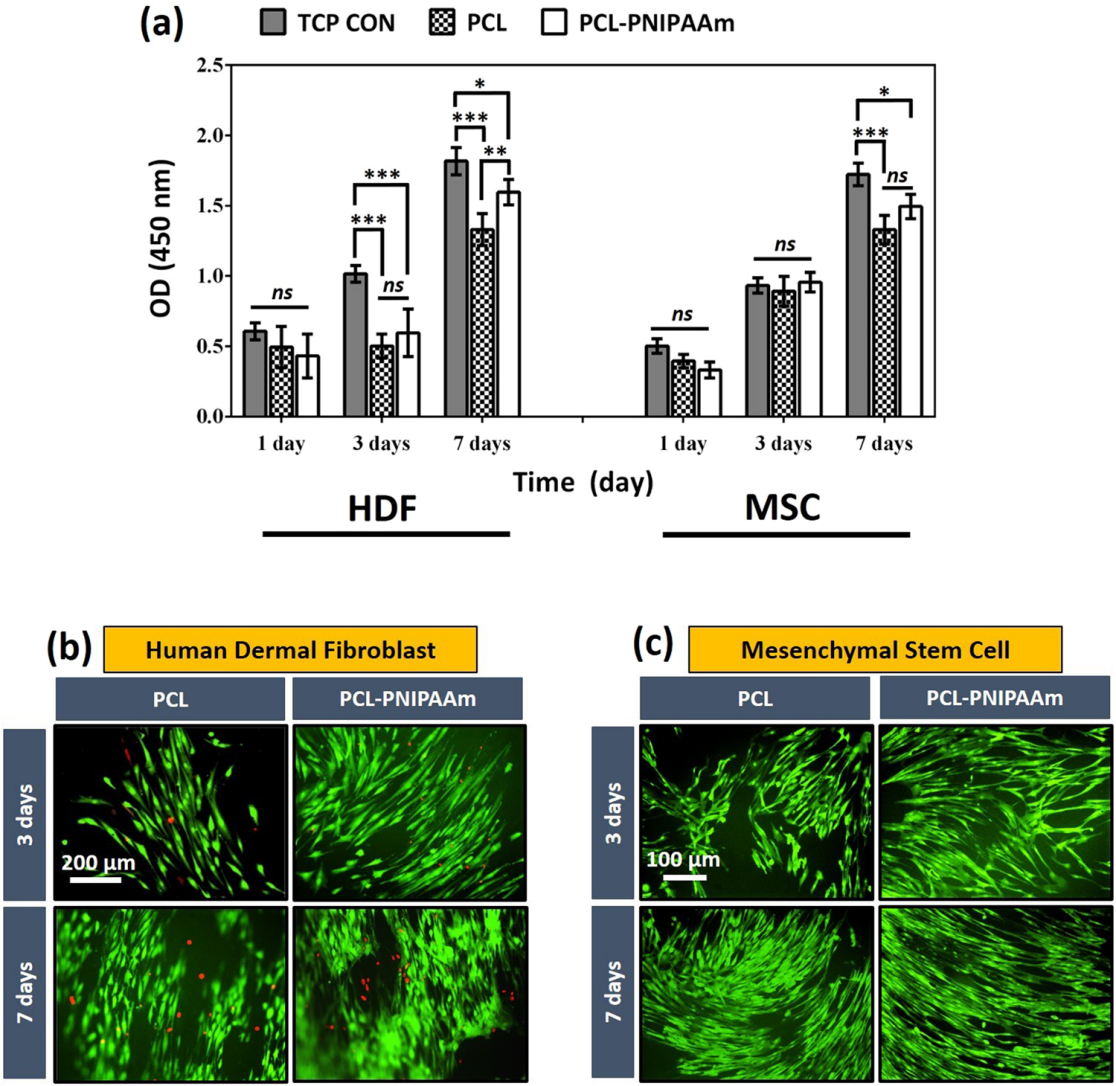
Cell proliferation on PCL and PCL-PNIPAAm surfaces. CCK-8 studies showed that both HDF cells and MSCs survived and proliferated on both PCL and PCL-P (PCL-PNIPAAm) using tissue culture plates as control (TCP CON) surfaces for 1, 3 and 7 days (**a**). At day 7, a significant increase in OD was observed in both cell types compared to day 1 and 3 and both types proliferated more on the surfaces of PCL-P than the surfaces of PCL (*p < 0.05; **p < 0.01; ***p < 0.001; ns: no significant difference). This trend of cell viability and proliferation was also observed in fluorescent microscopy for both HDF cells (**b**) and MSCs (**c**). HDF cells were stained with Live-dead staining (green: membrane of living cells, red: nuclei of dead cells) whereas GFP was cloned into MSCs. Emission was observed with fluorescent microscopy at days 3 and 7. Very dense and clustered cells with higher viability were observed at day 7 and on modified surfaces (PCL-P) than at day 3 and on non- modified PCL surfaces.

The PCL-PNIPAAm macrocarriers, which were 2.0 g of PNIPAAm-NH_2_ immobilised PCL, were used for further experiments. In order to assess whether the immobilised material and other chemical reactions of the conjugation procedure caused any adverse effect on cell growth and viability, we used CCK-8 assay for viability up to 7 days and observed cell growth both on PCL and PCL-PNIPAAm surfaces using fluorescent microscopy. Although compared to the control both PCL and PCL-PNIPAAm surfaces showed reduced viability, a consistent increase in cell proliferation on both surfaces was depicted as shown in Fig. 3. In addition, a slightly higher proliferation rate was observed in conjugated surfaces compared to the pure PCL (*p < 0.05 at day 7) (Fig. 3a).

Live-Dead images of HDF cells grown on PCL and PCL-PNIPAAm surfaces at 3 and 7 days of incubation are shown in Fig. 3b. The images show that most of the cells are live (green), although a few dead cells (red) were spotted on both surfaces. In addition, the morphology of the cells expressed the standard spindle-shaped morphology of fibroblast cells indicating that the cells were healthy and spreading on the macrocarriers. GFP was cloned into MSCs and green emission was observed with fluorescent microscopy at days 3 and 7. Very dense and clustered cells with higher viability were observed at day 7 and on conjugated surfaces (PCL-P) than at day 3 and pure PCL surfaces. Notably both cells were viable and showed their normal physiology and spindle shaped appearance. Altogether these results suggested that PNIPAAm immobilised onto PCL surfaces was risk-free, thus providing a valuable tool for recovering large-scale cellular collections.

### Cell detachment from macrocarriers

**Figure 4:**
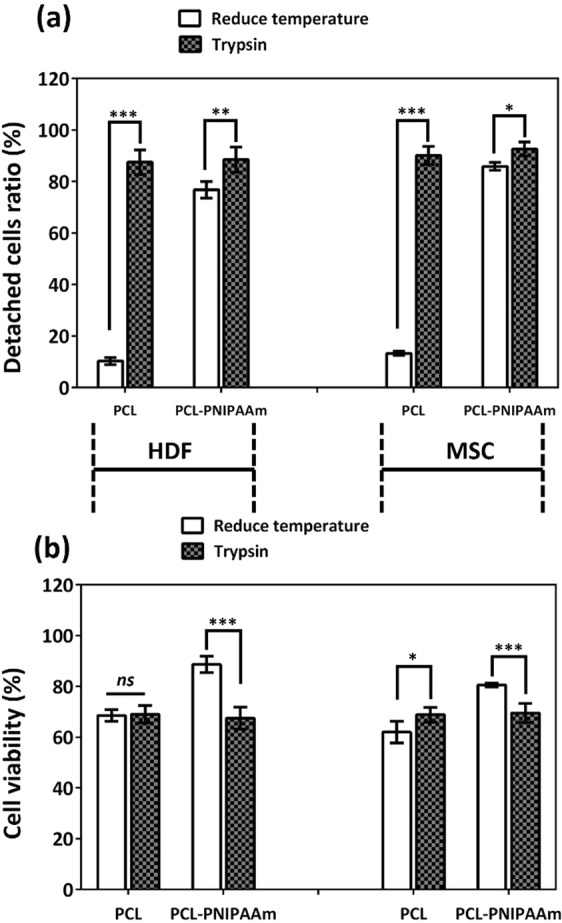
Trypsinization vs. reduced temperature comparison of cell detachment ratio and viability. Both HDF cells and MSCs in reduced temperature conditions (30 °C) showed significantly higher numbers of cell-detachment ratios from PCL-PNIPAAm surfaces than from PCL only surfaces. Trypsinization did not affect significantly the cell-detachment ratios between the surfaces but this technique allowed more detachment of cells from the surfaces of both PCL and PCL-PNPAAm surfaces. However, a higher cell viability rate was observed in the temperature dependent cell recovery technique than the trypsinization technique. Both HDF and MSCs had higher cell viability when recovered from PCL-PNIPAAm than trypsinization. *p < 0.05; **p < 0.01; ***p < 0.001; ns: no significant difference.

In order to ascertain the efficiency of cell detachment by lowering the temperature to 30 °C, we compared the recovered cells in reduced temperature conditions with trypsinization conditions. Figure 4a shows that more than 70% of the cells were detached from PNIPAAm-conjugated PCL surfaces simply by lowering the temperature. Trypsinization had a slightly higher detachment rate than the reduced temperature technique of thermo-responsive polymer.

### Cell proliferation comparisons between cells detached from PCL-PNIPAAm by reduced temperature and trypsinization

**Figure 5:**
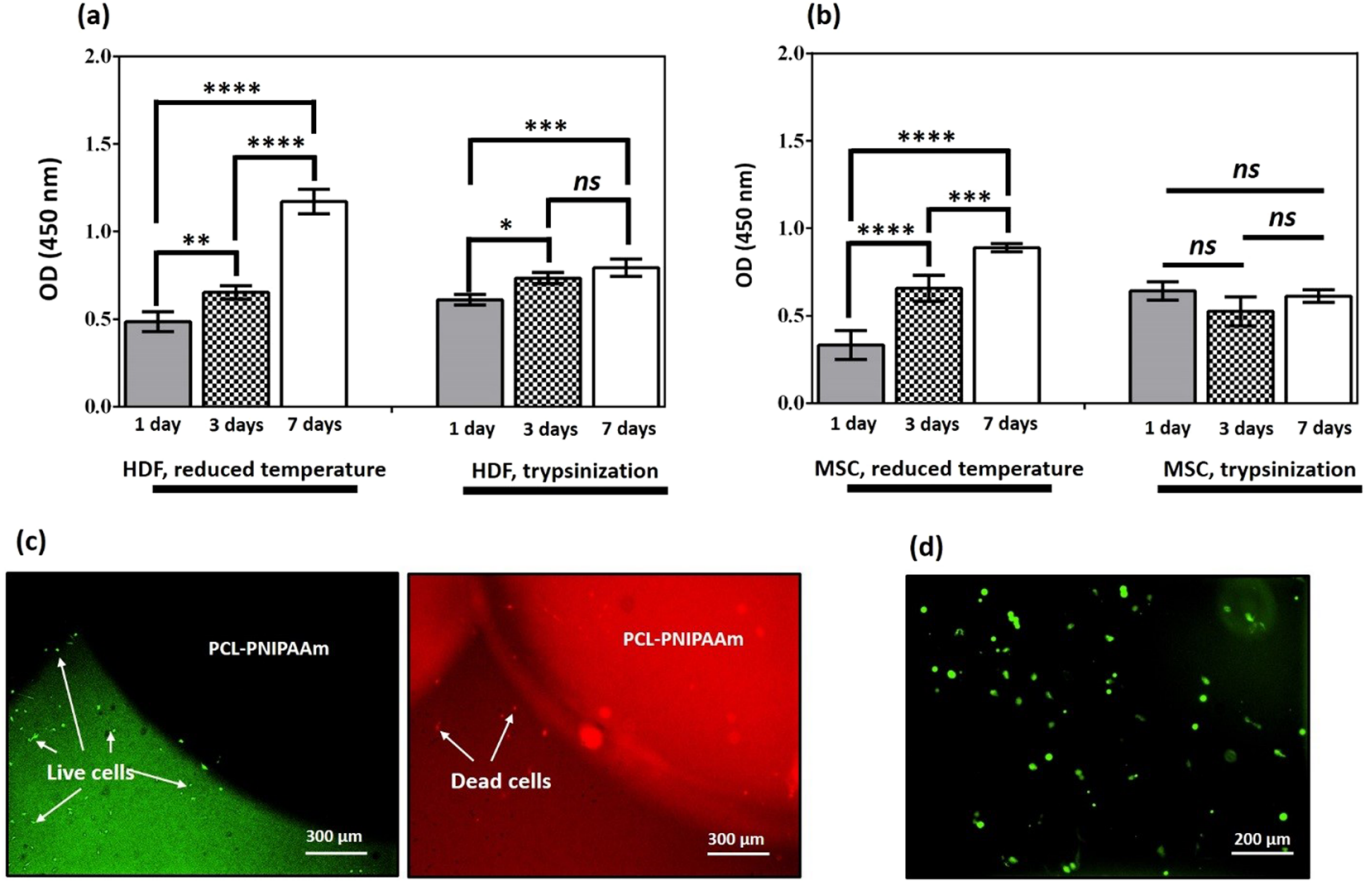
Cell proliferation comparisons between cells detached by reduced temperature and trypsinization. Both HDF cells (**a**) and MSCs (**b**) from PCL-PNIPAAm surfaces were recovered, collected and grown at 1, 3 and 7 days in culture media. CCK-8 studies showed that when both cell types were recovered from PCL-PNIPAAm surfaces using reduced temperature, cell growth was exponential and significant over time. However, when collected by trypsinization, cell growth was non-exponential and insignificant over time. Live-dead assay of the HDF cells propagated on the surfaces of PCL-PNIPAAm surfaces is shown in (**c**). A large number of live cells (green) were observed after 40 minutes of incubation at low temperature (30 °C). (**d**) Shows MSC after detachment from PCL-PNIPAAm by reduced temperature still strongly carrying GFP. *p < 0.05; **p < 0.01; ***p < 0.001; ****p < 0.0001; ns: no significant difference.

To demonstrate the propagation and proliferation into the immediate cellular passage of the harvested and recovered cells, a comparative viability assay was conducted for 1, 3 and 7 days between trypsin treated and recovered cells and reduced temperature and recovered cells (Fig. 5a,b). Both HDF and stem cells were used. After harvesting the cells either by using trypsin-EDTA or reduced temperature, equal numbers of cells were seeded for proliferation analysis. The results showed an exponential growth with a significant increase in cell numbers in both cell types collected from PCL-PNIPAAmm surfaces by reducing the temperature, although no significant increase was obtained between day 3 and 7 for HDF harvested with trypsin, and no proliferation at all for MSCs in the same conditions. These results clearly indicate that the cell recovery process involving the reduced temperature process was more efficient than the Trypsin-EDTA recovery process.

Live-dead assays of the cells propagated on the surfaces of PCL-PNIPAAmm surfaces are shown in Fig. 5c. A large number of live cells (green) were observed after 40 minutes of incubation at low temperature (30°C). On the other hand, GFP-labelled stem cells were also observed in fluorescence microscopy and a strong green florescence emitted from live cells was noticed. Both cases indicated a healthy cellular propagation on PCL-PNIPAAmm surfaces.

### ECMs on cells detached from PCL-PNIPAAm macrocarriers

**Figure 6:**
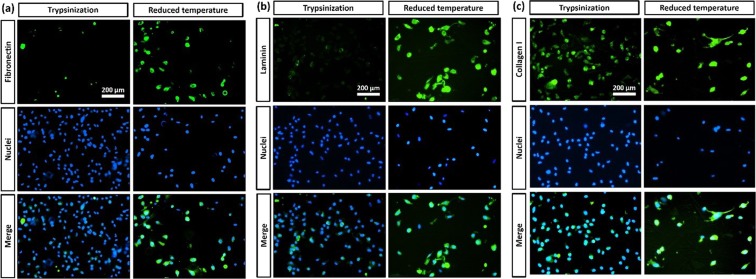
Immunofluorescent analysis. Representative Immunostaining results for ECM proteins of HDF cells. (**a**) Fibronectin, (**b**) Laminin and (**c**) Collagen I on cells detached from PCL-PNIPAAm macrocarriers by trypsin treatment and reduced temperature. Nuclei stained blue with Hoechst 33342 dye and proteins stained green with FITC-labelled secondary antibody. Immunofluorescent images showed that cells detached by reduced temperature contained fibronectin whereas no fibronectin was found in cells detached by trypsin treatment.

Representative immunostaining results for ECM proteins including Fibronectin, Laminin and Collagen I of HDF cells on cells detached from PCL-PNIPAAm macrocarriers by trypsin treatment and reduced temperature are shown in Fig. 6. Nuclei stained blue with Hoechst 33342 dye and proteins stained green with FITC-labelled secondary antibody. For the suspended cells harvested by trypsinization, only a small amount of Fibronectin and Laminin was distributed around the cell membranes with weak fluorescence. When treated by reduced temperature, HDF detached with strong green fluorescence evident of a large amount of Fibronectin and Laminin. The Collagen I expressed by cells detached by both trypsinization and by reduced temperature suggested that the Collagen I in the cell membranes was not effected by trypsinization.

**Figure 7:**
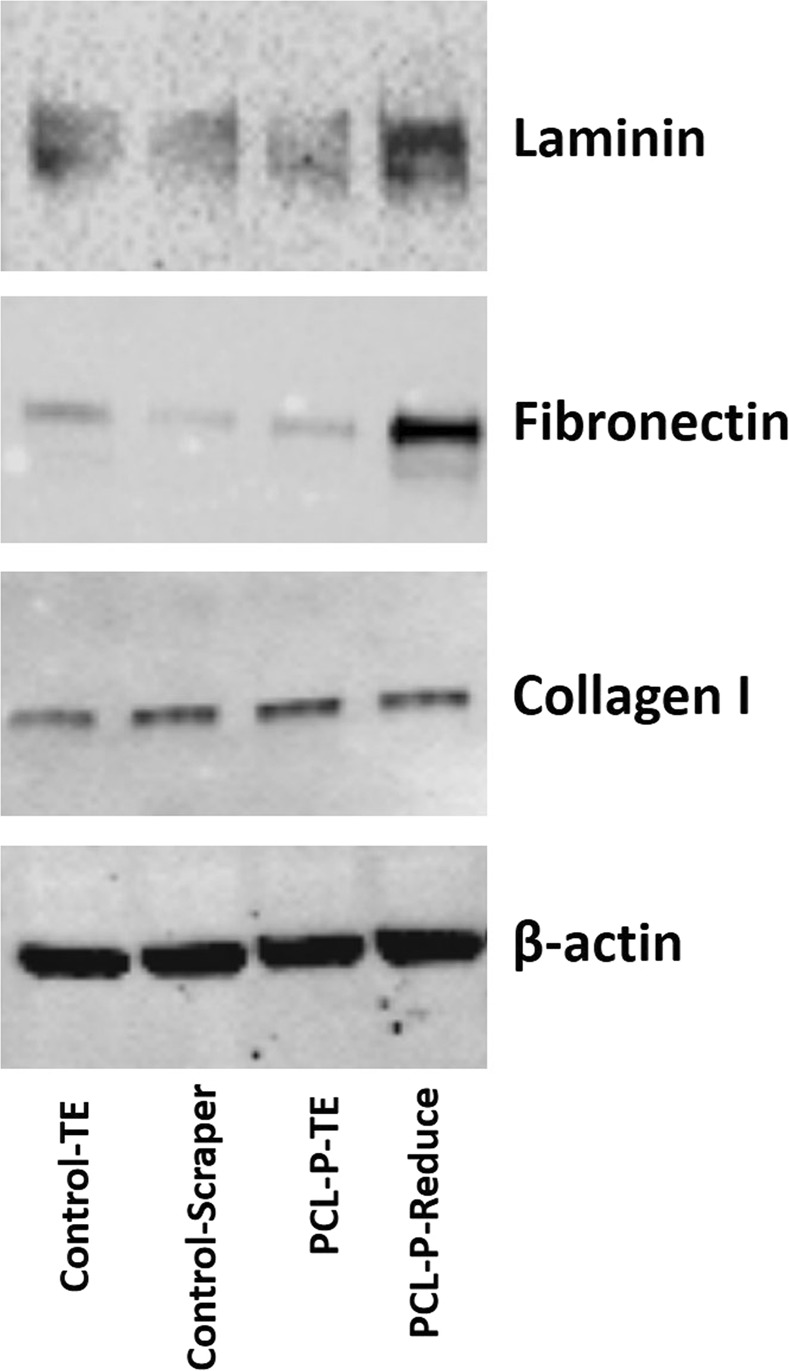
Western Blot analysis. Total proteins were collected from cells grown in tissue culture plates by harvesting the total cells using either trypsin-EDTA (Control-TE) or by scraping the monolayer of cells with a scraper (Control-Scraper). Cells were also grown on PCL-P (PCL-PNIPAAm) beads and harvested either with trypsin-EDTD (PCL-P-TE) or by reducing the temperature (PCL-P-Reduce). Antibodies against laminin, Fibronectin and Col 1 were used to detect the expression of these proteins by Western Blot after harvesting the cells in four different ways. b-actin was the control protein. The blots were cropped from different gels. All gels are run in the same experiment conditions. Full-length blots of each tested protein are presented in supplementary information (Fig. S3).

The expression patterns of Fibronectin, Laminin and Collagen I in recovered HDF cells (after detachment and collection) were analysed by Western Blot as shown in Fig. 7. The Fibronectin and Laminin expression in cells detached from PCL-PNIPAAm by reduced temperature was higher than the cells detached by trysinization. However, Collagen 1 was found to be equally expressed in cells and non-significantly affected by the two different processes. The result was consistent with the immunostaining data.

## Discussion

In this study, in order to immobilize the thermo-responsive polymer onto the surface of PCL beads, PNIPAAm-NH_2_ polymers were conjugated with PCL beads through amidation (“grafting-to” technique)^27^ between the carboxylate molecule on the PCL beads’ surface and the amine end group of PNIPAAm-NH_2_ (Fig. 1). In this case, a thin PNIPAAm brush layer is formed on the PCL surface, which determines the surface properties. PCL pellets were initially treated with NaOH, which causes the base hydrolysis of ester bonds in PCL to create carboxylate ions. The carboxyl functional groups on PCL beads can be activated by 1-ethyl-3-[3-dimethylaminopropyl]carbodiimide hydrochloride (EDC) and N-hydroxysuccinimide (NHS) to form succinimide esters, which in turn spontaneously react with the amine groups on the PNIPAAm-NH_2_. The reaction of carboxylate with EDC produces an unstable reactive O-acylisourea ester. Sulfo-NHS is then added to stabilize the intermediate ester, which then converts the unstable O-acylisourea into an amine-reactive NHS ester. This ester will react with the amino end of the PNIPAAm-NH2 to form a stable covalent amide bond between the PCL beads and the PNIPAAm-NH_2_.

As described earlier, the “grafting-to” approach was applied for the conjugation of PCL and PNIPAAm. This technique relies on the covalent tethering between preformed end-functionalized PNIPAAm and complementary reactive groups located on carrier surfaces, leading to strong binding^28^ between PCL surface and the PNIPAAm “coating”^26,29^. However, this definition could be considered too narrow, since useful grafting can also be achieved when appropriate functionality is located mid-chain. The “grafting-to” approach is widely used because it is experimentally simple and easy to control^29,30^. However, the grafting density obtained through this approach is limited due to steric hindrance caused by the already grafted polymer chains. Polymer chains have to diffuse through the existing polymer layer to reach the complementary reactive groups on the microsphere surface before they can be immobilised^26^.

The PNIPAAm conjugated PCL did not affect cell attachment and proliferation on the macrocarriers. In addition, the cell recovery process involving the reduced temperature process was more efficient than the Trypsin-EDTA recovery process. Trypsinization had a higher detachment rate than reducing the temperature of the thermo-responsive polymer. This was not surprising; other research groups have also reported higher detachment rates with enzymatic digestion than with the thermal reduction technique. However, the physiological damage caused by trypsin or other enzymes is the major reason why researchers wish to avoid enzymatic digestion in clinical applications. We showed here that the recovery potential of cells (HDF and MSC) after harvesting was significantly higher when the thermo-responsive method was used compared to trypsin. In another recent study, we also discussed the effects on the gene expression in stem cells in 3D culture, on our PCL-PNIPAAm macrocarriers among other 2D and 3D cultures^31^. The transcriptome of bone marrow MSCs cultured on our PCL macrocarriers showed consistent gene expression as compared with MSCs cultured in 2D tissue culture plastic surfaces^31^. Taken together, these results highlighted the functionality of MSCs after culture and harvesting on these thermo-responsive macrocarriers.

The fate of the expression of ECM proteins on cells detached from PCL-PNIPAAm by either trypsin or reduced temperature was observed by both immunostaining and immunoblotting. In this study, we seeded HDF on tissue culture plates and PCL-PNIPAAm, incubated for 7 days, and then collected the cells by either trypsin-EDTA treatment or simply by reducing the temperature. Cells were then immunostained or subjected for total protein collection for Western Blotting. Three major structural ECM proteins, Fibronectin (FN), Laminin (LM) and Collagen type I (Col I), were observed. They are the most abundant and important ECM proteins found in the cells/tissues and play various roles in foetal development, tissue repair and angiogenesis^32^. Because of their ‘glue-like’ properties, these proteins usually contribute to cell attachment to surfaces. Here we studied these proteins to see whether their expression was affected by the process employed to recover the cells. Although trypsin is widely used to harvest cells grown in 2D conditions with good viability and recovery, both immunostaining and immunoblotting results indicated here overall that trypsinization might adversely affect cell structure and physiology by degrading some of the structural ECM proteins when used on 3D cultures.

In contrast to microcarriers that are typically 100–300 µm in size, a larger particle size can present biological cells with a “flatter” surface upon which to adhere. As a result, the cells may be exposed to a gentler, more uniform, and optimal growing environment in a turbulent bioreactor system, with easier handling, collection and concentration steps This can help to improve the quality (e.g. viability and functionality) of the cells produced. Moreover, cell detachment from thermo-responsive macrocarriers in this study can be induced simply by reducing the temperature without the need for proteolytic enzyme treatment. Such thermally induced cell harvesting enhances the viability and therapeutic efficiency of harvested cells. This allows the cells to be recovered without the need, for example, of enzymatic treatments that can present an increased risk of cell damage.

Large-scale expansion of cells with consistent phenotype for clinical use is required. In addition, harvesting the cells from the carriers while maintaining the cells’ critical quality attributes is very different from monolayer culture and therefore poses a significant challenge. Thermo-responsive macrocarrier cell culture systems may provide the solution to such challenges by generating large-scale cell cultures in both clinical and industrial applications such as tissue engineering, cell transplantation and biological production.

## Materials and Methods

### Materials

Polycaprolactone pellets (PCL, Mn 80,000), sodium hydroxide (NaOH), 1-ethyl-3-[3-dimethylaminopropyl]carbodiimide hydrochloride (EDC), N-hydroxysuccinimide (NHS), morpholinoethanesulphonic acid (MES) and Poly (N-isopropylacrylamide), amine terminated average Mn 2500 (T) (PNIPAAm-NH_2_) were purchased from Sigma-Aldrich (UK) and used as received. The deionised water used in this study was obtained from an ultrapure water purification system (Elix®, Millipore).

### Preparation of PCL-PNIPAAm

The PCL pellets were immersed in NaOH 1 M solution for 1 h with constant shaking to obtain carboxylate ions PCL-COO^−^, and then they were rinsed with autoclaved deionized water 5 times.

Reaction buffer was prepared by dissolving 0.12 M EDC (0.46 g) and 0.06 M NHS (0.14 g) in 20 mL of 0.05 M MES buffer solution (pH 6). PNIPAAm-NH_2_ solutions were prepared by dissolving different masses of PNIPAAm-NH_2_ powder (0.2 g, 0.5 g, 1.0 g, 2.0 g, 3.0 g or 4.0 g) in 20 mL of deionized water to measure the respective C:O:N ratios (Table 1).

PCL-PNIPAAm macrocarriers were synthesized by conjugating PCL-COO^−^ pellets with PNIPAAm-NH_2_ through amidation reaction. First, 3.0 g of PCL-COO^−^ pellets were activated in the reaction buffer for 3 h at room temperature, then added to one of the PNIPAAm-NH_2_ solutions and gently shook at 4 °C overnight. PCL-PNIPAAm macrocarriers were then centrifuged at 1500 rpm for 10 min, washed five times with deionized distilled water, and lyophilized for 2 days.

### Characterizations and measurements

Fourier transform infrared (FTIR) spectra were recorded using an FTIR spectrometer (Bruker, Tensor 27) equipped with attenuated total reflectance (ATR, Pike). Before collecting sample spectra, the background spectrum was collected by measuring the response of the spectrometer without sample.

The surface morphology of the PCL and PCL-PNIPAAm macrocarriers was observed by SEM (Carl Zeiss Evo LS15 VP-Scanning Electron Microscope SE, BSE, VPSE, EPSE detectors) at an accelerating voltage of 10 kV. Before the SEM investigation, the samples were coated with gold by sputtering. INCA X-Act X-ray system (Oxford Instruments) and OIM XM 4 Hikari EBSD System (EDAX) were used for EDS analysis.

X-ray photoelectron spectroscopy (XPS) spectra for PCL and PCL-PNIPAAm were obtained at base pressure (1 × 10^−9^ torr) with variable aperture 3–10 mm and the data were analysed using CasaXPS peak fitting software.

### Cell culture on thermo-responsive macrocarriers

#### Sterilisation process

PCL and PCL-PNIPAAm macrocarriers were placed in a laminar hood and UV radiation was applied for 30 minutes. The samples were then turned over and irradiated for an additional 30 minutes. They were then immersed in 70% ethanol for 3 hours and washed with phosphate buffered saline (PBS, pH 7.4). Finally, the samples were immersed in cell culture medium (DMEM) for 3 hours in glass bottles before cell seeding. Glass bottles were siliconized with Sigmacote® prior to use to prevent cells adhering to the bottle walls.

#### Cell culture and seeding

Human dermal fibroblast cells (HDF, ThermoFisher Scientific) were cultured in Dulbecco’s modified Eagle’s medium (DMEM 4.5 mg/l of glucose; Gibco BRL, Gaithersburg, MD, USA) supplemented with 10% (v/v) foetal bovine serum (FBS; Gibco BRL) and 1% (v/v) penicillin-streptomycin (PS; Gibco BRL). Green Fluorescence Protein (GFP)-producing MSCs were kindly provided by the Department of Paediatrics and Adolescent Medicine, LKS Faculty of Medicine, The University of Hong Kong. Cell were pre-cultured in T flasks, harvested and seeded on the macrocarriers at the initial density of 3.2 × 10^5^ cells/ml (*i*.*e*. 9.6 × 10^5^ cells per bottle containing a monolayer of macrocarriers). Cell culture plates were also seeded with the same number of cells per sample (6-well plates) as control groups.

#### Cell viability, cytotoxicity and proliferation assessment

Cell viability, cytotoxicity and proliferation were determined by CCK-8 assay (Sigma) for 1, 3 and 7 days of incubation and Live-Dead assay using fluorescein diacetate (FDA; Sigma) and ethidium bromide (EB, Sigma) for 3 and 7 days of incubation. FDA stained the cytoplasm of viable cells green while EB stained nuclei of non-viable cells red.

To measure the efficiency of cell attachment to macrocarriers within 24 h of culture, the macrocarrier-free supernatant was carefully removed and the number of cells in the supernatant was determined with a haemocytometer. The number of cells attached to macrocarriers was calculated by subtracting the number of cells in the supernatant from the total cell number at inoculation. The attachment yield was calculated as follows:

Attachment yield (%) = (number of cells attached to macrocarriers/total number of cells number at inoculation) × 100.

#### Cell detachment from macrocarriers

After 1 day of suspension culture, either trypsin treatment or temperature change was applied to the samples.

For thermo-responsive detachment, the temperature of the culture medium was reduced from 37 °C to 30 °C by using a dedicated incubator. Cells cultured on two types of macrocarriers (PCL or PCL-PNIPAAm) were incubated for 40 min at 30 °C. The number of detached cells in the supernatant was counted with a haemocytometer.

For trypsin detachment, culture medium was first removed from samples before washing with 37 °C PBS and immersing in trypsin-EDTA for 5 minutes at 37 °C. Trypsin was then inactivated by adding fresh medium, cell suspension was homogenised and the number of detached cells was counted with a haemocytometer.

Cell detachment ratio = [number of detached cells]/[total number of attached cells on macrocarriers before detachment] × 100.

#### Extra cellular matrix (ECM) protein expression

ECM protein expression of HDF was analysed by immunofluorescence staining and Western Blot analysis. After 7 days of culture, HDF were detached from PCL-PNIPAAm macrocarriers by either reduced temperature or trypsin as described earlier and fixed with 4% (w/v) paraformaldehyde for 15 min at room temperature. Cells grown on tissue culture plates were also harvested using either trypsin-EDTA (Control-TE) or by scraping the monolayer of cells with a scraper (Control-Scraper) to be used as controls.

To detect fibronectin, laminin and collagen I on the detached cells with immunofluorescence staining, anti-fibronectin (1:200, Santa Cruz, Biotechnology, CA, USA), anti-laminin (1:200, Santa Cruz, Biotechnology, CA, USA) and anti-collagen I (1:50, Santa Cruz, Biotechnology, CA, USA) antibodies were used overnight at 4 °C. The cells were then incubated with a secondary antibody (Alexa Flour 488, 1:1000, Invitrogen). The images were taken using a cooled CCD camera (EXI blue; Olmaging, UK) attached to an inverted microscope (Eclipse Ti, Nikon).

For Western Blot analysis, detached cells were rinsed and treated with lysis buffer (RIPA, Millipore, Milford, MA, USA), vortexed on ice 5 times within 20 min and centrifuged for 10 min at 13,000 rpm at 4 °C. Aliquots (30 µg) of the proteins were analysed by Western Blot. The dilution of 1:100 of anti-fibronectin (Fibronectin (EP5): sc-8422, Santa Cruz Biotechnology, INC.), anti-laminin (Laminin β-2 (C4): sc-59980, Santa Cruz Biotechnology, INC.) and anti-collagen type I (COL1A (COL-1): sc-59772, Santa Cruz Biotechnology, INC.) antibodies were used as proteins for the analysis. Anti-beta actin antibody (1:500 dilution of β-Actin (C4): sc-47778, Santa Cruz Biotechnology, INC.) was used as a control. The proteins were separated by 4–20% SDS-PAGE gels (Mini-PROTEAN TGX Precast Protein Gels, Bio-Rad) and electroblotted onto polyvinylidene difluoride membranes (Immun-Blot PVDF Membrane, Bio-Rad). The membranes were blocked with 5% fat-free milk at room temperature for 30 min before incubation with primary antibodies overnight at 4 °C. The immunocomplexes were visualized using enhanced chemiluminescence reagent.

### Statistical analysis

All quantitative data were expressed as mean ± SD of independent replicates. Statistical analysis was performed with two-way analysis of variance (ANOVA) with Tukey’s honest significant difference post hoc test. All analyses were carried out using GraphPad Prism 6 with a value of *p < *0.05 considered statistically significant.

## Conclusion

In this study, we successfully immobilized PNIPAAm onto PCL macro-bead surfaces. Cell viability and cytotoxicity studies confirmed that the newly formed beads were non-toxic, risk-free and biocompatible with human dermal fibroblast cells and mesenchymal stem cells. Cell attachment onto the surfaces was also confirmed. We also successfully showed that just by reducing the temperature from 37 °C to 30 °C, more than 70% of the cells were collected without the need for physical force or enzyme treatment. More importantly, cells recovered well from detachment with a higher proliferation rate than with trypsin-based harvesting. ECM protein expression for cells detached from macrocarriers by temperature reduction compared to trypsin treatment confirmed the potential of thermo-responsive macrocarriers for future use in large-scale cell recovery.

## Supplementary information

Supplementary Information
