# Supplementary material for: Development of thermo-responsive polycaprolactone macrocarriers conjugated with Poly(N-isopropyl acrylamide) for cell culture
Source: Sci Rep. 2019 Mar 5;9:3477. doi: 10.1038/s41598-019-40242-0 (PMC6401373; doi:10.1038/s41598-019-40242-0)

Supplementary Information for *Scientific Reports*

Development of thermo-responsive polycaprolactone macrocarriers conjugated with Poly(N-isopropyl acrylamide) for cell culture

Linh T. B. Nguyen[1], Akinlolu O. O. Odeleye[1,†], Chih–Yao Chui[1], Timothée Baudequin[1], Zhanfeng Cui[1], Hua Ye [1,*]

[1]*Institute of Biomedical Engineering, Department of Engineering Science, University of Oxford, OX3 7DQ, UK.*

[†]*Current Address: Adaptimmune Limited, 60 Jubilee Avenue, Milton Park, Abingdon, OX14 4RX, UK*

Supplementary Figure S1

Figure S1. (a) Hoechst staining images of HDF on PCL-PNIPAAm after 7 days of incubation and (b) GFP was cloned into MSCs and green emission was observed in a fluorescent microscopy at day 3 and 7 (in low magnification).

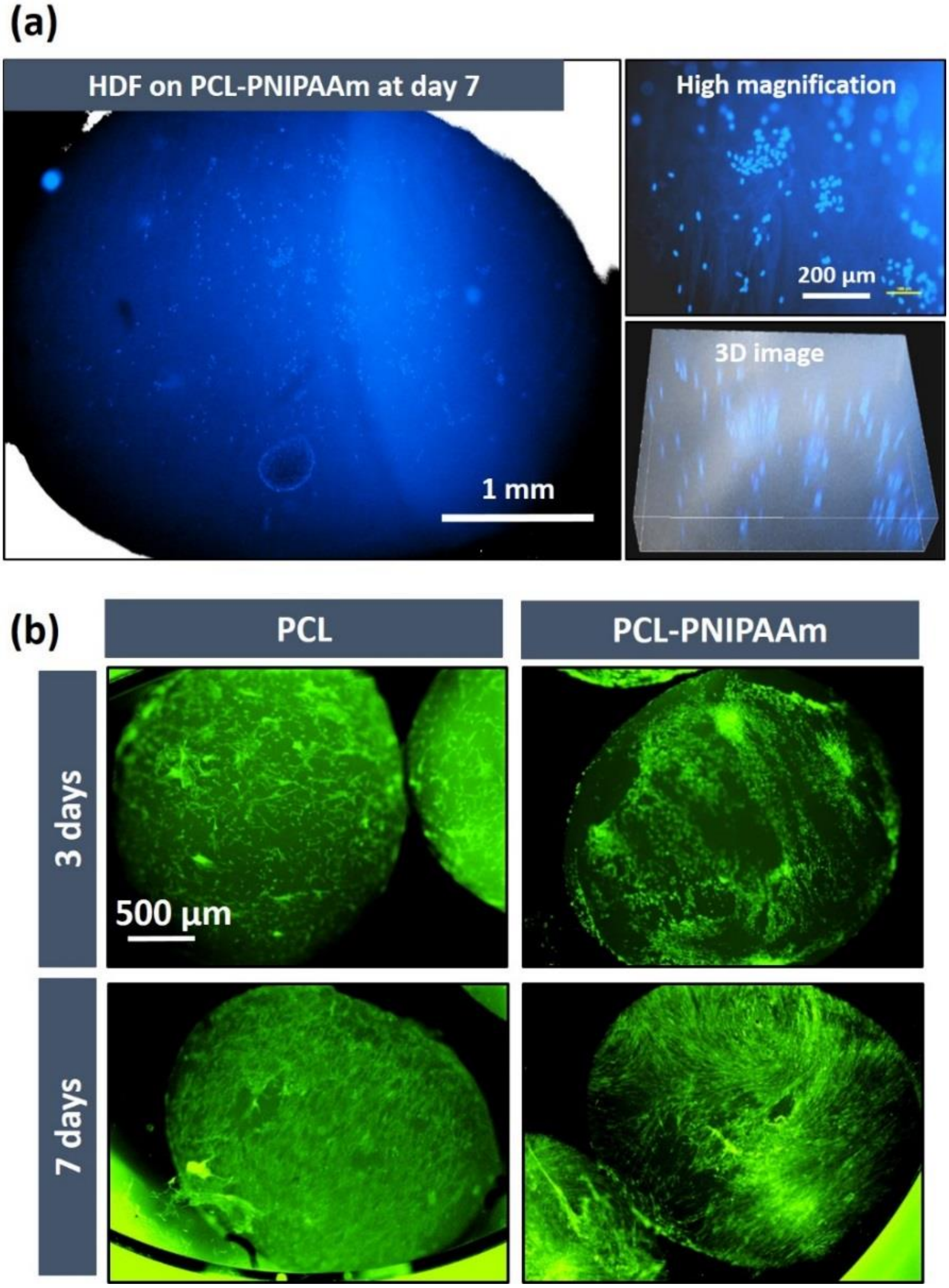

Supplementary Figure S2

Figure S2. (a) and (b) Phase contrast images of HDF detachment from PCL and PCL-PNIPAAm by either trypsin treatment or temperature drop from 37 °C to 30 °C and (c) Green field fluorescence images of MSC detachment from PCL and PCL-PNIPAAm by either trypsin treatment or temperature drop from 37 °C to 30 °C.

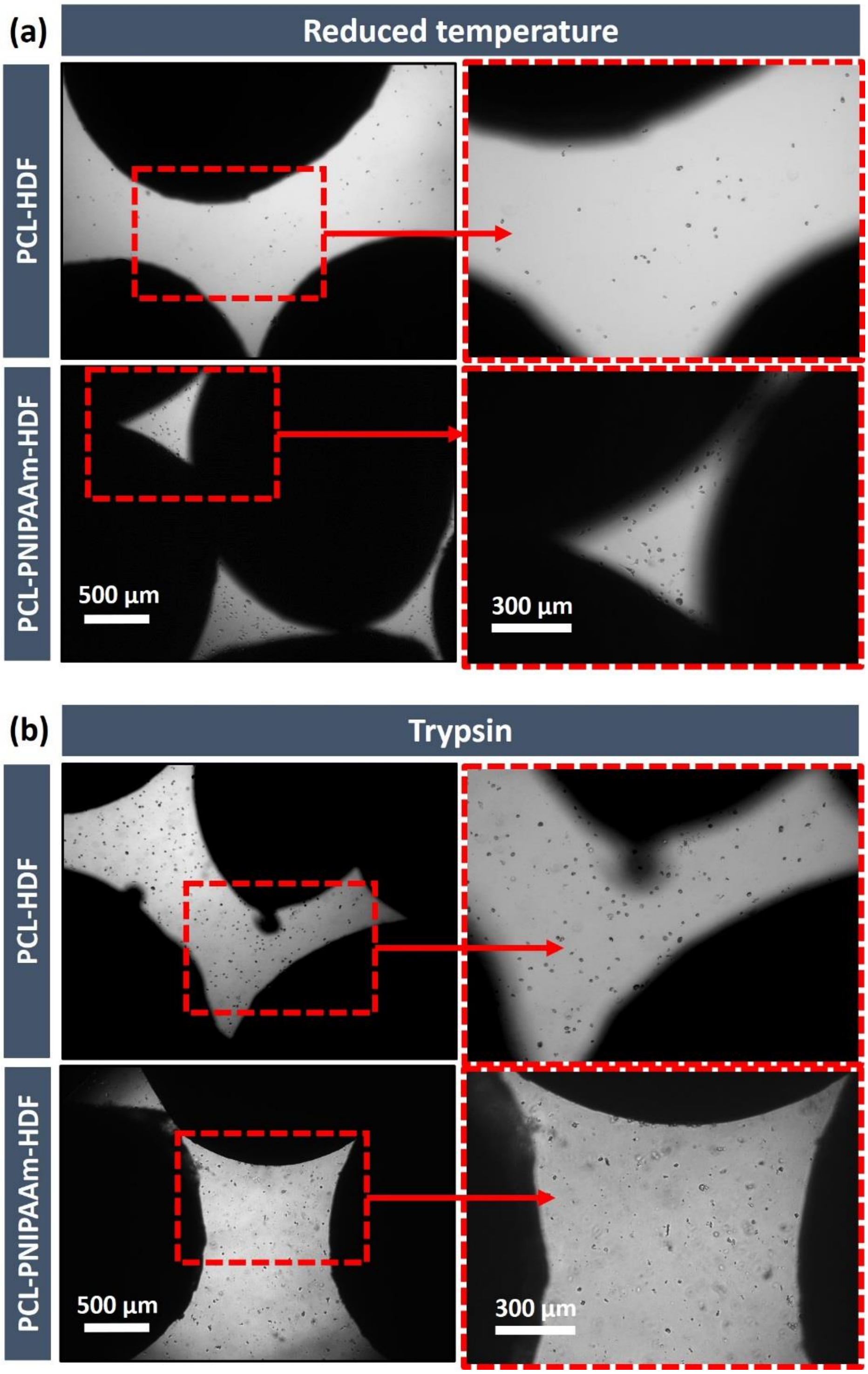

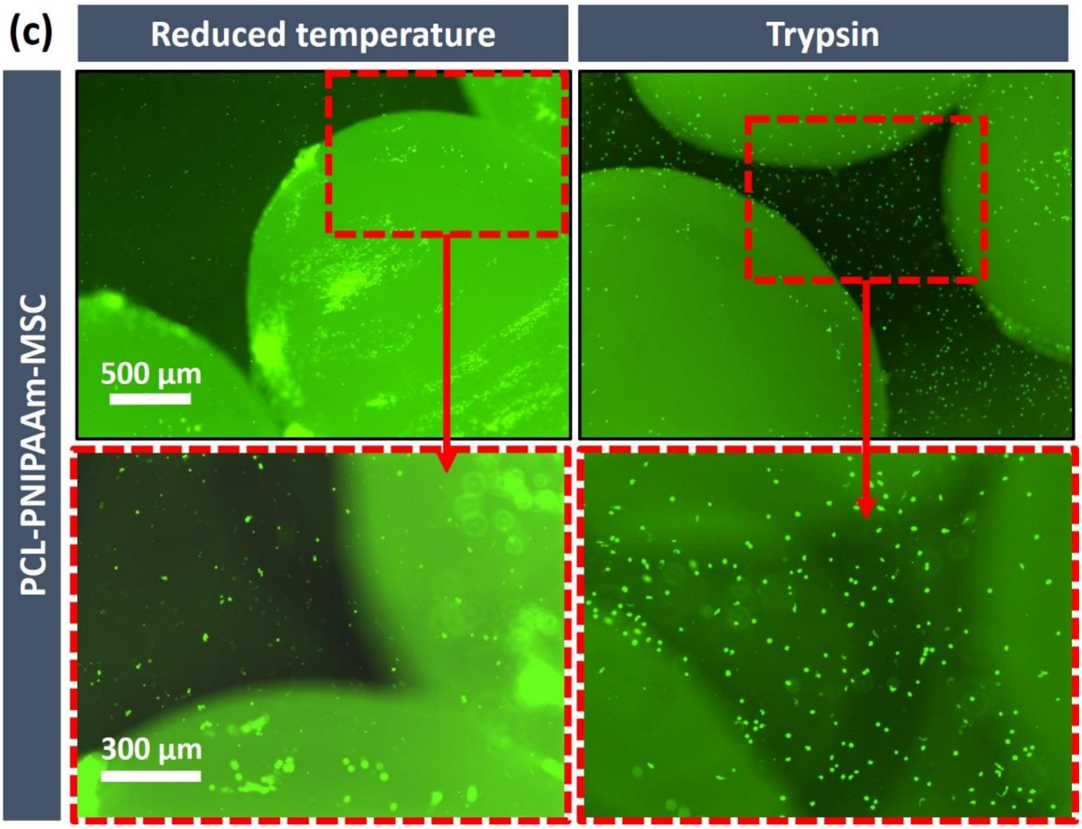

(c)
Reduced temperature
Trypsin
PCL-PNIPAAm-MSC
500 µm
300 µm

Supplementary Figure S3

Figure S3. Full-length Western Blots: (a) Antibodies against laminin (lane 1-4), (b) Fibronectin (lane 1-4), (c) Collagen 1 (lane 1-4) and beta-actin (lane 1-4). Marker: lane 0 (Precision Plus Protein™ Kaleidoscope™ Prestained Protein Standards, Bio-Rad).

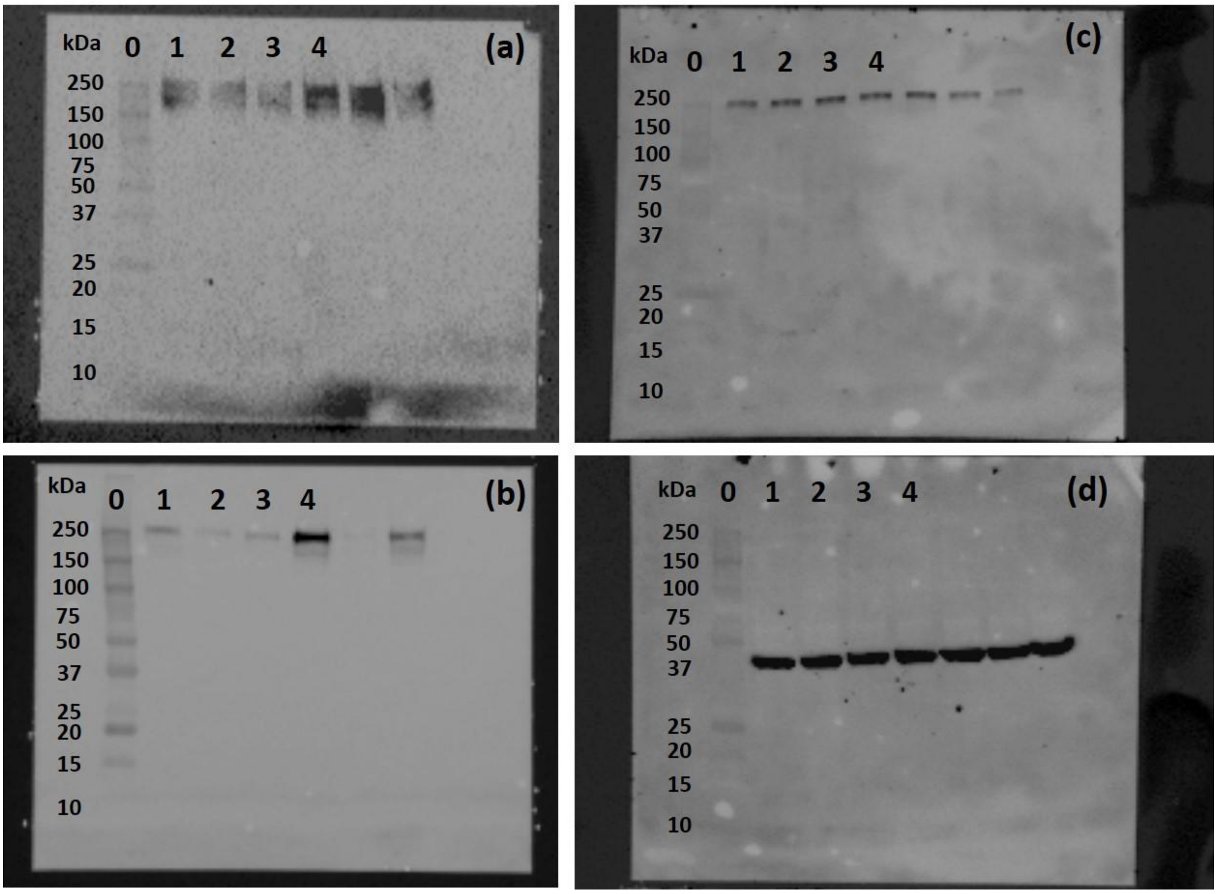

Supplementary Figure S4

Figure S4. ATP- FTIR analysis of PCL-PNIPAAm after 1, 3 and 7 days immersed in culture media at 37 °C. The results showed that the peaks at 1565 cm^{-1} corresponded to the amide II bond and 1647 cm^{-1} corresponded to the amide I bond were still present after 7 days, confirming that the conjugation was still stable after one week in culture conditions.

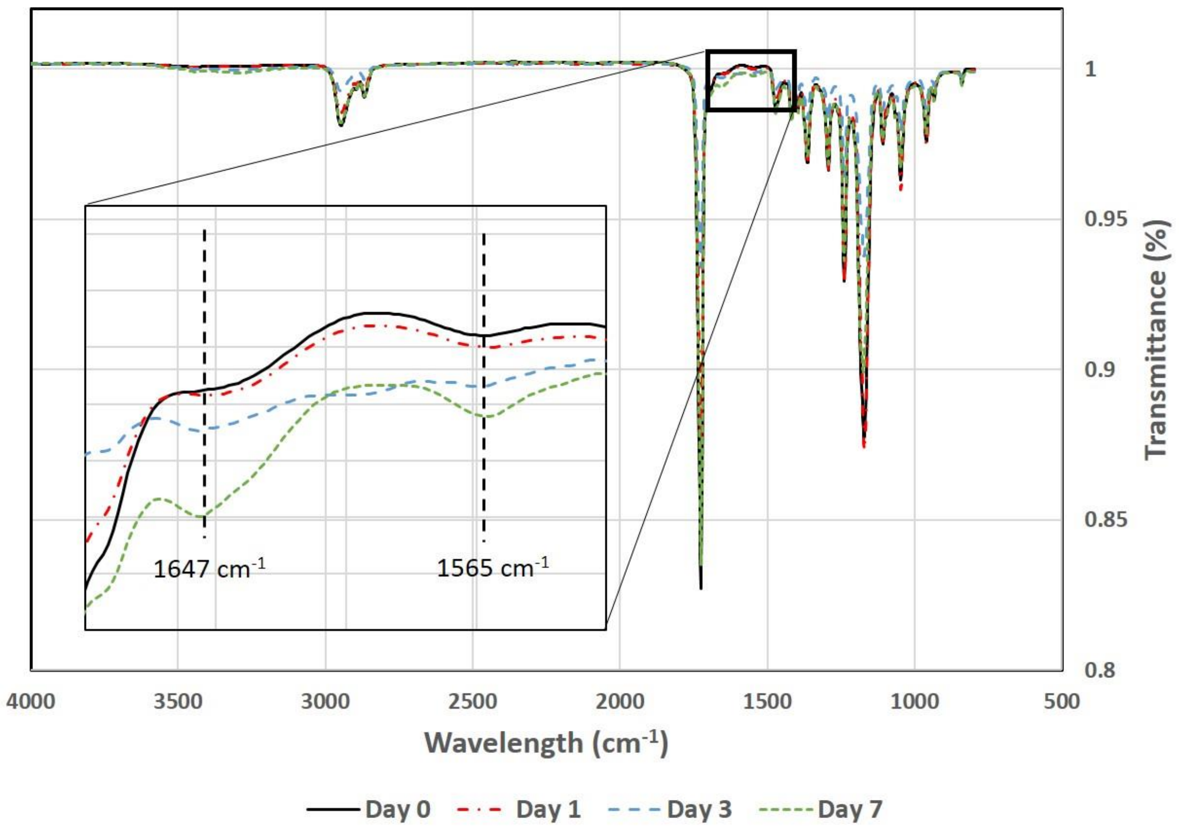

Supplement: Supplementary file 1 — Supplementary Information [file 41598_2019_40242_MOESM1_ESM.pdf]
